# A preclinical evaluation of pemetrexed and irinotecan combination as second-line chemotherapy in pancreatic cancer

**DOI:** 10.1038/sj.bjc.6603726

**Published:** 2007-04-10

**Authors:** A Mercalli, V Sordi, R Formicola, M Dandrea, S Beghelli, A Scarpa, V Di Carlo, M Reni, L Piemonti

**Affiliations:** 1 Laboratory of Experimental Surgery, San Raffaele Scientific Institute, Via Olgettina 60, Milan 20132, Italy; 2 Section of Anatomic Pathology, Department of Pathology, University of Verona, Strada Le Grazie 8, Verona 37134, Italy; 3 Department of Oncology, San Raffaele Scientific Institute, Via Olgettina 60, Milan 20132, Italy

**Keywords:** pancreatic cancer, chemiosensitivity, human

## Abstract

Gemcitabine (GEM)-based chemotherapy is regarded as the standard treatment of pancreatic adenocarcinoma, but yields a very limited disease control. Very few studies have investigated salvage chemotherapy after failure of GEM or GEM-containing chemotherapy and preclinical studies attempting to widen the therapeutic armamentarium, not including GEM, are warranted. MIA PaCa2, CFPAC-1 and Capan-1 pancreatic cancer cell lines were treated with GEM, fluouracil (5-FU), docetaxel (DCT), oxaliplatin (OXP), irinotecan (CPT-11), pemetrexed (PMX) and raltitrexed (RTX) as single agent. Pemetrexed, inducing apoptosis with IC_50_s under the *C*_max_ in the three lines tested, appeared the most effective drug as single agent. Based on these results, schedule- and concentration-dependent drug interactions (assessed using the combination index) of PMX/GEM, PMX/DCT and PMX–CPT-11 were evaluated. The combinatory study clearly indicated the PMX and CPT-11 combination as the most active against pancreatic cancer. To confirm the efficacy of PMX–CPT-11 combination, we extended the study to a panel of 10 pancreatic cancer cell lines using clinically relevant concentrations (PMX 10 *μ*M; CPT-11 1 *μ*m). In eight of 10 lines, the PMX–CPT-11 treatment significantly reduced cell recovery and increased both the subG1 and caspase 3/7 fraction. After a 5-day wash out period, an increased fraction of subG1 and caspase3/7 persisted in PMX–CPT-11-pretreated cell lines and a significant reduction in the clonogenicity capacity was evident. Finally, *in vivo*, the PMX/CPT-11 combination showed the ability to inhibit xenograft tumours growth as second-line therapy after GEM treatment. The PMX and CPT-11 combination displays a strong schedule-independent synergistic cytotoxic activity against pancreatic cancer, providing experimental basis for its clinical testing as salvage chemotherapy in pancreatic cancer patients.

Until a decade ago, the use of chemotherapy in pancreatic cancer was believed to have no role in the routine treatment of patients with advanced disease (Lionetto *et al*, 1995). To date, some options are available for first-line treatment (Burris *et al*, 1997; Reni *et al*, 2005). Single-agent fluorouracil (5-FU) results in tumour response rates of 7% or less (Cullinan *et al*, 1990; Burris *et al*, 1997). Combination chemotherapy with 5-FU has resulted in increased toxicity without higher efficacy (Schnall and Macdonald, 1996; Ducreux *et al*, 2002; Maisey *et al*, 2002; Beger *et al*, 2003). Gemcitabine (GEM) in weekly infusions has been shown to be superior to bolus 5-FU as monotherapy for advanced disease in a randomised phase III study and was licensed for treatment of advanced pancreatic cancer (Burris *et al*, 1997). Also a comprehensive experience reported in a large, multicentre, open-label study that enrolled more than 3000 patients on a compassionate-need basis documented single-agent GEM to be reasonably safe and to offer a median overall survival of 4.8 months (Storniolo *et al*, 1999). So GEM is currently regarded as the standard treatment for advanced disease, and has led to an objective response in 4–26% of patients and a 1-year overall survival of 17–28% of patients in phase III trials (Burris *et al*, 1997; Bramhall *et al*, 2001; Berlin *et al*, 2002; Bramhall *et al*, 2002; Heinemann, 2002; Moore *et al*, 2003; Rocha Lima *et al*, 2004; Van Cutsem *et al*, 2004; Reni *et al*, 2005).

Despite the fact that randomised studies have suggested that chemotherapy is superior to best supportive care in prolonging survival and improving symptoms in patients with advanced disease, standard single-agent GEM yields a marginal impact on disease outcome. Several attempts to improve the efficacy of GEM in advanced pancreatic adenocarcinoma by addition of a second cytotoxic agent or other drugs to a standard dose and schedule of GEM have not shown a significant survival advantage (Burris *et al*, 1997; Bramhall *et al*, 2001; Berlin *et al*, 2002; Bramhall *et al*, 2002; Heinemann, 2002; Moore *et al*, 2003; Rocha Lima *et al*, 2004; Van Cutsem *et al*, 2004; Mishra *et al*, 2005; Reni *et al*, 2005; Stathopoulos *et al*, 2006). GEM-based chemotherapy yields a very limited disease control, and progression usually occurs within a few months after first-line treatment starts. Median progression-free survival with single-agent GEM is approximately 3 months and less than 15% of patients are progression free at 6 months from diagnosis (Burris *et al*, 1997; Bramhall *et al*, 2001; Berlin *et al*, 2002; Bramhall *et al*, 2002; Heinemann, 2002; Moore *et al*, 2003; Rocha Lima *et al*, 2004; Van Cutsem *et al*, 2004; Reni *et al*, 2005). In spite of progressive disease, about half of the patients maintain a good performance status and are willing to undergo further treatment. So far very few studies have investigated salvage chemotherapy after failure of GEM or GEM-containing chemotherapy (Oettle *et al*, 2000; Ulrich-Pur *et al*, 2003; Cantore *et al*, 2004; Milella *et al*, 2004; Reni *et al*, 2004). As no standard therapeutic option exists and scarce information on the impact on outcome of salvage therapy is available from the literature, preclinical studies attempting to widen the therapeutic armamentarium, not including GEM, are warranted.

The present study was performed in pancreatic cancer lines to identify GEM-free combination of drugs. The PMX and CPT-11 combination showed a strong schedule-independent synergistic cytotoxic activity against pancreatic cancer, providing experimental basis for its clinical testing as salvage chemotherapy in pancreatic cancer patients.

## MATERIALS AND METHODS

### Drug and chemical

GEM and PMX were from Eli Lilly (Indianapolis, IN, USA), RTX was from AstraZeneca (Basiglio, Italy), OXP from Sanofi Synthelabo (Milan, Italy), DCT and CPT-11 from Aventis (Milan, Italy), 5-FU from Teva Pharma Italia (Milan, Italy). Drugs were dissolved in sterile distilled water and diluted in culture medium immediately before use.

### Drug pharamacokinetics

The data on the pharmacokinetics of the drugs (*C*_max_) in human were obtained by the revision of the earlier publications (Abbruzzese *et al*, 1991; Burris *et al*, 1993; Extra *et al*, 1993; Pazdur *et al*, 1993; Rothenberg *et al*, 1993; Clarke *et al*, 1996; Chabot, 1997; Hui and Reitz, 1997; Beale *et al*, 1998; Gamelin *et al*, 1998; Judson *et al*, 1998; O'Dwyer *et al*, 1999; Rinaldi, 1999; Culy *et al*, 2000; Jodrell *et al*, 2001).

### Cell culture

Pancreatic cancer cell lines MIA PaCa2, CFPAC-1, HS766T, T3M4, A818-4 (American Type Culture Collection, Manassas, VA, USA), Capan-1, PaCa3, SK-PC 1, PANC-2, PC (generous gift from Professor A Scarpa, University of Verona, Verona, Italy) were cultured as monolayers in RPMI 1640 (Biochrom, Berlin, Germany), supplemented with 10% heat-inactivated FCS (Hyclone, Logan, UT, USA), penicillin and streptomycin (100 *μ*g ml^−1^) under standard culture conditions (5% CO_2_, 95% air in humidified chamber at 37°C). Cells were cultivated in 72 cm^2^ flasks (Costar, Cambridge, MA, USA) and were harvested with trypsin when they were in logarithmic growth.

### Cell cycle, apoptosis analysis, clonogeneic capacity

Cells were plated in 24-well sterile plastic plates (Costar) at 15 × 10^4^ cell ml^−1^ and were allowed to attach for 24 h. Then, cells were treated with different concentration (0.001–100 *μ*M) and combination of GEM, 5-FU, DCT, OXP, CPT-11, PMX and RTX for 48 h. After drug treatment, cells were trypsinised, washed once with PBS, and fixed with 70% ethanol at −20°C for 24 h. Fixed cells were washed three times and stained with a propidium iodide (PI; Sigma Chemical Co., St Louis, MO, USA) solution (20 *μ*g ml^−1^) containing 0.1 mg ml^−1^ of RNase A (Sigma Chemical Co., St Louis, MO, USA). Cells were then subjected to cell cycle analysis for determining DNA contents by flow cytometry (FACScan, Cell Quest software; BD Biosciences, San Jose, CA, USA). Cell debris was excluded on the basis of forward *vs* side scatter. Doublets and clumps were excluded by gating on a bivariate distribution of AUX (PI peak pulse) *vs* the PI-integrated signal. Data from 10 000 events were collected in the final gated histograms. Apoptotic cells were identified on the basis of hypodiploid DNA content (subG_1_ fraction) that results from DNA fragmentation and confirmed with the evaluation of phosphatidylserine exposure using annexin V-FITC Kit (Bender MedSystems, San Bruno, CA, USA) in combination with PI.

The activation of caspase 3/7 was analysed after drug treatment using Carboxyfluorescein FLICA Assay Kits (B-Bridge International, Inc., Sunnyvale, CA, USA) according to the manufacturer's instructions.

For the clonogenicity assay, cells were exposed for 48 h to the drug; then cells were washed, plated by limiting dilution down to three cell per well and cultured with drug-free fresh medium. After 1 week, each well was checked by optical microscopy for growing colonies (at least four cells per well).

The IC_50_ was defined as the drug concentration required to induce 50% of apoptotic cells and was calculated by nonlinear least-square curve fitting. Drug interaction was assessed at different concentration ratio (0.1 : 1; 1 : 1; 10 : 1) using the combination index (CI; Chou *et al*, 1994), where CI<1, CI=1 and CI>1 indicate synergistic, additive and antagonistic effects, respectively.

Since several preclinical studies have shown a schedule-dependent drug interaction for the antimetabolites in combination regimens (Tolis *et al*, 1999; van Moorsel *et al*, 1999; Peters *et al*, 2000; Edelman *et al*, 2001; Symon *et al*, 2002; Giovannetti *et al*, 2004), we tested, *in vitro,* different treatment schedules. For the experiments of sequential exposure, cells were treated with (a) drug 1 (0.001–100 *μ*M) for 48 h; (b) drug 2 (0.001–100 *μ*M) for 48 h; (c) drug 1 together with drug 2 (0.001–100 *μ*M, ratio 1 : 1) for 48 h; (d) drug 1 (0.001–100 *μ*M) for 24 h followed by drug 2 for 24 h (ratio1 : 1); (e) the reverse sequence of point (d). On the basis of the isobologram analysis for mutually exclusive effects, the CI value was calculated as follows:

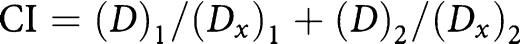

where (*D*_*x*_)_1_ and (*D*_*x*_)_2_ are the concentrations of the single drugs required to induce cell apoptosis by 50%, and (*D*)_1_ and (*D*)_2_ are the drug concentrations in combination treatments which also induce cell apoptosis by 50% (isoeffective as compared with single drugs). In some experiments, a nonconstant ratio combination design was used and the CI value for each data point was calculated. Data analysis was performed by the Calcusyn Software (Biosoft, Oxford, UK).

## *IN VIVO* STUDY

MIA PaCa2, CFPAC-1 and PaCa3 cells (5 × 10^6^ cells for each mice) were s.c. injected into female nude mice (4 weeks of age, Harlan, Italy). One week after cell inoculation (day 0), five randomised animals for each experimental group received GEM (150 mg kg^−1^ i.p.) at day 0, +3, +6, +9, +12, +15, +18 and +21 or GEM (150 mg kg^−1^ i.p.) at day 0, +3, +6 and +9 and then PMX (100 mg kg^−1^ i.p.) every day starting from day +12 until day +21 plus CPT-11 (50 mg kg^−1^ i.p.) at day +12 and +17. Control group received only the vehicle (PBS i.p.) at the same time. Tumour volume and body weight were daily recorded for each animal for all the period of drug treatments (day 0–21) and for 10 days after treatment suspension (days 22–32). Tumour volume was calculated using the formula: *V*=*π*/6 × (largest diameter × smallest diameter)^3/2^. The ethical standards of the experiment were approved by the Verona University Review Board, and the animals were maintained in accordance with institutional guidelines.

### Statistical analysis

Data were expressed as mean values +s.d. or median (25–75° percentiles). Differences between the IC_50_ were analysed by Wilcoxon Signed Ranks test. The potential of drugs for inhibition of *in vivo* tumour growth was analysed using Tukey's HSD test. Statistical analyses were performed using the Statistical Package for Social Science (SPSS 11.0; SPSS, Chicago, IL, USA).

## RESULTS

### Induction of apoptosis by GEM, 5-FU, DCT, OXP, CPT-11, PMX and RTX in pancreatic cancer cell lines: a comparative evaluation in relation with the ‘*in vivo*’ plasma maximum concentration (*C*_max_) of the single drugs

**Figure 1:**
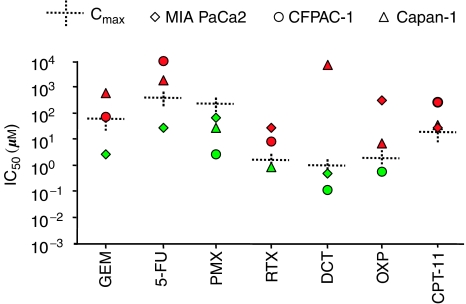
Drug concentrations required to induce the 50% apoptotic cells (IC_50_) in culture. MIA PaCa2, CFPAC-1 and Capan-1 pancreatic cancer cell lines were treated with gemcitabine (GEM), fluorouracil (5-FU), docetaxel (DCT), oxaliplatin (OXP), irinotecan (CPT-11), pemetrexed (PMX) and raltitrexed (RTX) for 48 h with different concentration (0.001–100 *μ*M). Data are expressed as median of 12 experiments. The plasma maximum concentration of the drugs (*C*_max_) as described by human pharmacokinetics studies (Abbruzzese *et al*, 1991; Burris *et al*, 1993; Extra *et al*, 1993; Pazdur *et al*, 1993; Rothenberg *et al*, 1993; Clarke *et al*, 1996; Chabot, 1997; Hui and Reitz, 1997; Beale *et al*, 1998; Gamelin *et al*, 1998; Judson *et al*, 1998; O'Dwyer *et al*, 1999; Rinaldi, 1999; Culy *et al*, 2000; Jodrell *et al*, 2001) was also reported. Red colour-filled symbol: IC_50_>*C*_max_; green colour-filled symbol: IC_50_< *C*_max_.

MIA PaCa2, CFPAC-1 and Capan-1 pancreatic cancer cell lines were treated with GEM, fluouracil (5-FU), docetaxel (DCT), oxaliplatin (OXP), irinotecan (CPT-11), pemetrexed (PMX) and raltitrexed (RTX) as single agent. The efficacy of the drugs was evaluated considering the 50% induction apoptosis concentration (IC_50_) in relation with the *C*_max_ (Figure 1). The majority of the IC_50_s measured resulted abundantly over the *C*_max_s of the drugs, confirming pancreatic cancer as a chemoresistant tumour. A dose-dependent induction of apoptosis with IC_50_ under the *C*_max_ in all the three lines tested was observed only by using PMX (*C*_max_ 229 *μ*M; median IC_50_: 67.16, 2.54 and 27.12 *μ*M, respectively, for MIA-PaCa2, CFPAC-1 and Capan-1; *n*=12). Two lines of three showed IC_50_ under *C*_max_ in the presence of DCT (*C*_max_ 0.99 *μ*M; median IC_50_: 0.48, 0.1147 and 8109 *μ*M, respectively, for MIA-PaCa2, CFPAC-1 and Capan-1; *n*=12). One line of three showed IC_50_ under *C*_max_ in the presence of RTX (*C*_max_ 1.54 *μ*M; median IC_50_: 27.77, 7.37 and 0.86 *μ*M, respectively, for MIA-PaCa2, CFPAC-1 and Capan-1; *n*=12), OXP (*C*_max_ 2.05 *μ*M; median IC_50_: 312, 0.57 and 6.58 *μ*M, respectively, for MIA-PaCa2, CFPAC-1 and Capan-1; *n*=12), GEM (*C*_max_ 61 *μ*M; median IC_50_: 2.49, 72.48 and 559 *μ*M, respectively, for MIA-PaCa2, CFPAC-1 and Capan-1; *n*=12) and 5-FU (*C*_max_ 372 *μ*M; median IC_50_: 26.19, >10 000 and 1750 *μ*M, respectively, for MIA-PaCa2, CFPAC-1 and Capan-1; *n*=12). Finally, no lines showed IC_50_ under *C*_max_ in the presence of CPT-11 (*C*_max_ 16.05 *μ*M; median IC_50_: 25.64, 232 and 30.33 *μ*M, respectively, for MIA-PaCa2, CFPAC-1 and Capan-1; *n*=12).

### Study of PMX/GEM, PMX/DCT and PMX/CPT-11 combination

We chose PMX as the drug to be tested in combination studies, since it appeared the most efficient drug in inducing apoptosis as single agent. We assessed the interaction of PMX with GEM (antimetabolite), DCT (antimicrotubule agent) and CPT-11 (topoisomerase inhibitors) in a constant ratio combination experimental design. We used three different concentration ratios for the study (10 : 1; 1 : 1; 0.1 : 1).

**Figure 2:**
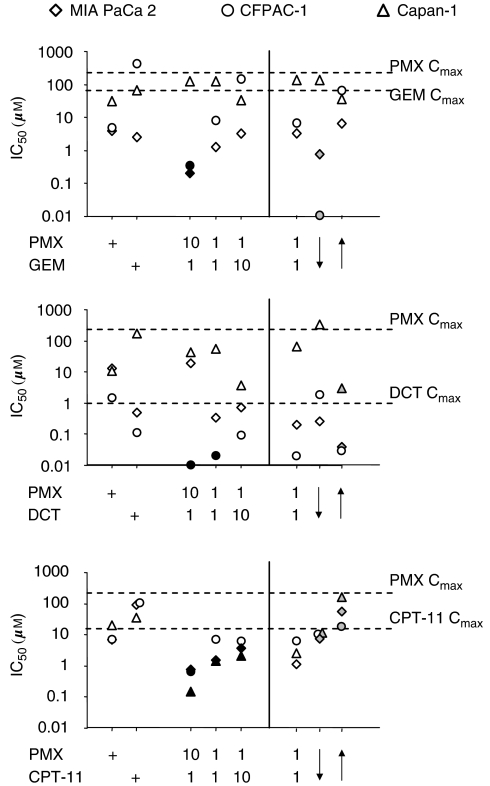
The IC_50_s (*μ*M) of PMX–GEM, PMX–DCT and PMX–CPT-11 combinations. The 10 : 1, 1 : 1 and 1 : 10 concentration ratio of PMX/GEM, PMX/DCT and PMX/CPT-11 combinations were represented. Moreover, MIA PaCa2, CFPAC-1 and Capan-1 pancreatic cancer cell lines were treated with (a) PMX together with GEM, DCT or CPT-11 (0.001–100 *μ*M, ratio 1 : 1) for 48 h; (b) PMX (0.001–100 *μ*M) for 24 h followed by GEM, DCT or CPT-11 for 24 h (ratio1 : 1); (c) the reverse sequence of point (b). The IC_50_ values of the different ratio and sequences were calculated and reported. All the values are expressed as medians of six experiments. Black colour-filled symbol=IC_50_ significantly less than the IC_50_s of the single drug (*P*<0.05, Wilcoxon Signed Ranks test). Grey colour-filled symbol=IC_50_ significantly less than the IC_50_ of the simultaneous exposure for 48 h (*P*<0.05, Wilcoxon Signed Ranks test). Dotted lines: *C*_max_ of GEM, DCT, CPT-11 and PMX.

**Figure 3:**
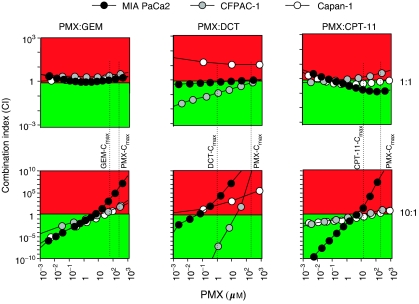
Combination index (CI) plots of PMX/GEM, PMX/DCT and PMX/CPT-11 association in MIA PaCa2, CFPAC-1 and Capan-1 pancreatic cancer cell lines. Data are represented as concentration/combination index (CI) plot. The CI was calculated in a constant ratio combination experimental design. The 1 : 1 and 10 : 1 concentration ratio of PMX/GEM, PMX/DCT and PMX/CPT-11 combinations were represented. CI<1 (green colour), CI=1 and CI>1(red colour) indicate synergistic, additive and antagonistic effects, respectively. Dotted lines: *C*_max_ of GEM, DCT, CPT-11 and PMX. The plots represent the mean of six experiments.

The simultaneous exposure to PMX and CPT-11 appeared the most efficient combination in inducing apoptosis with IC_50_ less than the IC_50_s of the single drugs in all the lines tested (Figure 2). The effect appeared particularly strong at the 10 : 1 ratio (but it is also present at 1 : 1 and 1 : 10 ratio) and the calculation of the CI showed synergism at effect levels obtainable with concentration under the *C*_max_ of CPT-11 and PMX (Figure 3).

The PMX–GEM combination appeared efficient in inducing apoptosis (IC_50_ less than the IC_50_ of the single drugs) in two of three lines tested at the 10 : 1 ratio concentration (Figure 2), and the CI values at the same ratio showed synergism at concentrations under the *C*_max_ of GEM and PMX (Figure 2). At 1 : 1 ratio concentration, an additive (MIA PaCa2, Capan-1) or antagonistic (CFPAC-1) effect was present (Figure 3).

Finally, the effect of DCT in combination with PMX was variable and cell line dependent (Figures 2 and 3). CFPAC-1 cell line appeared sensitive to the action of DCT–PMX combination (IC_50_ less than the IC_50_s of the single drugs at 10 : 1 and 1 : 1 ratios tested and strong synergism both at 10 : 1 and 1 : 1 ratios), while, on the contrary, Capan-1 appeared resistant (no reduction of IC_50_s at any ratio tested and CI indicating substantially an antagonism). MIA PaCa2 cell line showed an intermediate level of sensitivity.

**Figure 4:**
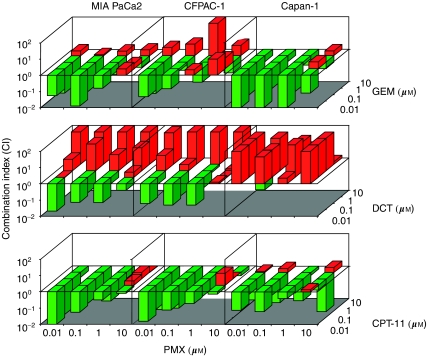
Combination index (CI) of PMX interaction with GEM, DCT and CPT-11 in a nonconstant ratio combination design. The concentrations of each drug from 0.01 to 10 *μ*M were tested. CI<1 (green colour), CI=1 and CI>1 (red colour) indicate synergistic, additive and antagonistic effects, respectively. The data represent the mean of three experiments.

The interaction of PMX with GEM, DCT and CPT-11 was also tested in a nonconstant ratio combination design varying the concentrations of each drug from 0.01 to 10 *μ*M. As long as the shape of the dose–effect curve and the median effect dose parameters for each single drug are available, the CI values for each data point of the nonconstant ratio design were calculated (Chou *et al*, 1994). The nonconstant ratio combination experiments confirmed the results of the constant ratio experiments and in particular the strong synergistic effect of PMX and CPT-11 in all the lines tested (Figure 4).

### Study of the schedule-dependent activity of PMX–GEM, PMX–DCT and PMX–CPT-11 combination

**Figure 5:**
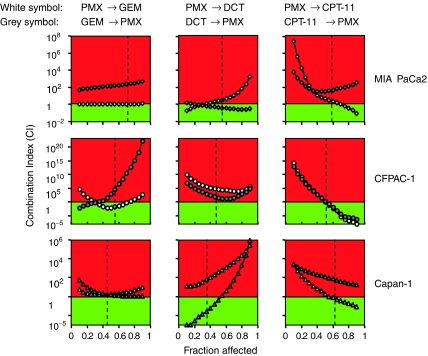
Combination index (CI) plots of schedule-dependent PMX/GEM, PMX/DCT and PMX/CPT-11 combination in MIA PaCa2, CFPAC-1 and Capan-1 pancreatic cancer cell lines. Cell lines were treated with (a) PMX (0.001–100 *μ*M) for 24 h followed by GEM, DCT or CPT-11 for 24 h at 1 : 1 ratio (white symbols); (b) the reverse sequence of point (a) (grey symbols). CI<1 (green colour), CI=1 and CI>1 (red colour) indicate synergistic, additive and antagonistic effects, respectively. The data represent the mean of three experiments. Dotted lines: fraction affected at *C*_max_ of different drug for each line tested.

Cell lines were treated with (a) PMX together with GEM, DCT or CPT-11 (0.001–100 *μ*M, ratio 1 : 1) for 48 h; (b) PMX (0.001–100 *μ*M) for 24 h followed by GEM, DCT or CPT-11 for 24 h (ratio1 : 1); (c) the reverse sequence of point (b). The IC_50_ and CI values of the different sequences were calculated (Figures 2 and 5).

The sequential exposure to PMX followed by GEM (PMX → GEM) appeared more efficient in inducing apoptosis than the reverse sequence (GEM → PMX) in CFPAC-1 and MIA PaCa2 lines, while this schedule of treatment appeared substantially indifferent in Capan-1 line (Figure 2). The CI calculation confirmed in CFPAC-1 line the synergism for PMX → GEM sequence at effect level obtainable with concentration under the *C*_max_ of GEM, while the GEM → PMX sequence produced antagonism in all the lines tested (Figure 5).

For the PMX–DCT combination, DCT followed by PMX (DCT → PMX) was more efficient in inducing apoptosis than the reverse sequence (PMX → DCT). DCT → PMX sequence reduced the IC_50_ under the level of simultaneous exposure in Capan-1 and MIA PaCa2 lines, while it did not induce any substantial modification in CFPAC-1 line (Figure 2). In Capan-1 and MIA PaCa2 lines, the calculation of CI value confirmed the synergism for DCT → PMX sequences (Figure 5).

Finally, the sequential exposure to PMX followed by CPT-11 (PMX → CPT-11) appeared more efficient than the reverse sequence (CPT-11 → PMX) in inducing apoptosis in all the lines, but the IC_50_ values of the PMX → CPT-11 sequence were higher than those of the simultaneous exposure (Figure 2). Moreover, the calculation of the CI value at effect level obtainable with concentration under the *C*_max_ of CPT-11 showed antagonism for both the PMX → CPT-11 and CPT-11 → PMX sequences in all the lines (Figure 5). These data suggested that for the PMX–CPT-11 combination, the sequential exposure to the drugs does not improve the action in comparison to the simultaneous exposure.

### Study of the PMX–CPT-11 combination activity on a large panel of pancreatic cancer cell lines: effect on cell survival, cell cycle, clonogenicity, caspases 3–7 activation

To confirm the efficacy of PMX–CPT-11 combination, we extended the study to a larger panel of pancreatic cancer cell lines using clinically relevant concentrations. Ten pancreatic cancer cell lines (PaCa3, SK-PC 1, PANC-2, MiaPaCa-2 from primary cancer; CFPAC1, PC, HS766T, Capan-1, T3M4, from metastases; A818-4 from ascites) were treated with the PMX (10 *μ*M)/CPT-11(1 *μ*M) combination for 48 h. Cell recovery, viability and apoptosis (cell cycle and caspase 3/7 activation) were analysed. In eight of 10 lines, PMX/CPT-11 significantly reduced cell recovery and increased both the subG1 and caspase 3/7 fraction (Table 1). To evaluate the cells that have entered into a necrotic or senescent phases during the treatment and that have lost the capacity to reproduce, after the 48 h drug exposure, the pancreatic cancer cell lines were washed and cultured in a drug-free environment for 5 days. After the wash out period, an increased fraction of subG1 and caspase 3/7 persisted in PMX–CPT-11-pretreated cell lines. Moreover, a significant reduction in the clonogenicity capacity of the lines was evident (Table 2).

### *In vivo* antitumour effect of PMX–CPT-11 combination after GEM treatment in xenografted nude mice

**Figure 6:**
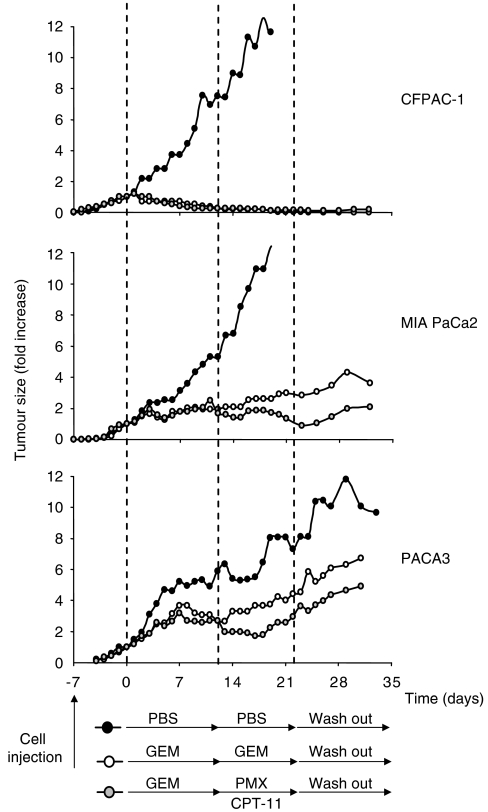
Antitumour activity of PMX/CPT-11 combination against pancreatic cancer xenografts. MIA PaCa2, CFPAC-1 and PACA3 xenograft tumours established subcutaneously in athymic nude mice were randomised to treatment groups (*n*=5 mice pergroup). The treatment groups were untreated controls (black symbol: PBS i.p.), GEM alone (white symbol: 150 mg kg^−1^ i.p. on days 0, +3, +6, +9, +12, +15, +18, +21) or PMX/CPT-11 after GEM treatment (grey symbol: GEM 150 mg kg^−1^ i.p. on days 0, +3, +6, +9; PMX 100 mg kg^−1^ i.p. on every day starting from day +12 until day +21; CPT-11 50 mg kg^−1^ i.p. on days +12 and +17) Data are expressed as the median change (fold increase) in tumour volume relative to volume on day 0, when treatment was initiated (MIA PaCa2=89.288 mm^3^; CFPAC-1=65.46 mm^3^; PACA3=261.87 mm^3^).

To evaluate the *in vivo* antitumour effect of PMX–CPT-11 combination as second-line salvage chemotherapy, CFPAC-1, MIA PaCa2 and PACA3 xenograft tumours established subcutaneously in athymic nude mice were treated with vehicle (PBS) or GEM (days 0–12). Starting from day +12, the GEM-treated tumours were randomised to be treated with PMX/CPT-11 (day 12–22) or with GEM (days 12–22) (Figure 6). The growth of CFPAC-1 xenografts was completely abolished by i.p. injection of GEM. In detail, at 12 days, the mean volumes were 32±24 and 29±11 mm^3^ in the two groups receiving GEM, which were significantly smaller than that in control group (516±133 mm^3^; *P*<0.001). The shift of the treatment to PMX/CPT-11 did not change the growth of CFPAC-1 in comparison to GEM. At 22 days, the mean volumes were 5±3 and 20±15 mm^3^, respectively, in groups receiving GEM or PMX–CPT-11 (*P*=0.89), which remained significantly smaller than that in control group (1304±458 mm^3^; *P*<0.001). The inhibition of the growth was maintained even after drug withdrawn. The growth of MIA PaCa2 xenografts was significantly inhibited, but not completely abolished by i.p. injection of GEM. In detail, at 12 days, the mean volumes were 194±92 and 178±29 mm^3^ in the two groups receiving GEM, which were significantly smaller than that in control group (468±151 mm^3^; *P*<0.01). The shift to the PMX/CPT-11 was more effective in inhibiting the growth of MIA PaCa2 than the maintenance of GEM treatment. At 22 days, the mean volumes were 315±135 and 138±33 mm^3^, respectively, in groups receiving GEM or PMX–CPT-11 (*P*=0.05) and both remained significantly smaller than that in control group (1122±334 mm^3^; *P*<0.001). Also in MIA PaCa2 xenografts, the effect on the growth was maintained even after drug withdrawn. Similarly to MIA PaCa2 xenografts, PACA3 xenografts showed a partial sensibility to the GEM action and an increased inhibition of growth after the shift to the PMX/CPT-11 treatment. At 12 days, the mean volumes were 866±273 and 770±216 mm^3^ in the two groups receiving GEM, which were significantly smaller than that in control group (1610±445 mm^3^; *P*<0.05). At 22 days, the mean volumes were 1322±440 and 798±171 mm^3^, respectively, in groups receiving GEM or PMX–CPT-11 (*P*=0.05) and both remained significantly smaller than that in control group (2147±463 mm^3^; *P*<0.01).

## DISCUSSION

The aim of the present study was to identify *in vitro* new drug combinations to be used in clinical testing as salvage chemotherapy in pancreatic cancer patients after GEM failure. The PMX and CPT-11 combination showed the strongest schedule-independent synergistic cytotoxic activity. Its efficacy was confirmed *in vitro* in a large panel of pancreatic cancer cell lines using clinically relevant concentrations and *in vivo* in three xenograft tumours providing experimental basis for its clinical testing as salvage chemotherapy in pancreatic cancer patients.

Pemetrexed inhibits thymidylate synthase, dihydrofolate reductase, and glycinamide ribonucleotide formyltransferase (Shih *et al*, 1997), thereby depleting nucleotide pools and blocking DNA synthesis (Tonkinson *et al*, 1997; Chen *et al*, 1999). Single-agent PMX *in vivo* has demonstrated activity in pancreatic cancer with a response rate of 5.7%, median survival of 6.5 months and 1-year survival of 28%, as reported in a phase II study (Miller *et al*, 2000; Kindler, 2002). On the basis of these data, together with phase I data showing synergy between GEM and PMX in a broad range of tumours, a phase III study of GEM/PMX combination was conducted on pancreatic cancer patients (Kindler, 2002; Oettle *et al*, 2005). Unfortunately, the results showed that the combination of PMX and GEM did not improve survival in patients with unresectable locally advanced or metastatic pancreatic cancer with an increase in toxicity compared with GEM monotherapy, but other combinations of PMX should continue to be explored in an effort to further improve the treatment of this chemorefractory disease.

Based on the PMX activity as single agent, we tested PMX in combination with DCT (antimicrotubule agent) and CPT-11 (topoisomerase inhibitors). The combinations of PMX with OXP or RTX were not assessed because a phase II clinical trial with RTX and OXP as salvage chemotherapy in GEM-resistant metastatic pancreatic cancer was already concluded and the results were recently reported (Reni *et al*, 2006). The *in vitro* study clearly indicates the PMX and CPT-11 combination as the most active one against pancreatic cancer: (i) the PMX and CPT-11 combination strongly reduced the IC_50_ in all the three lines tested; (ii) the effect was relevant at concentration that it is possible to achieve *in vivo*; (iii) the effect was maximum for the relevant clinical concentration ratio of 10 : 1. (iv) the sequential exposure PMX → CPT-11 appeared more efficient than the reverse sequence CPT-11 → PMX, but the IC_50_ values of the PMX → CPT-11 sequence remained higher than those of the simultaneous exposure.

The efficacy of PMX–CPT-11 combination was confirmed in a panel of 10 pancreatic cancer cell lines using clinically relevant concentrations (PMX 10 *μ*M; CPT-11 1 *μ*m). Based on the data obtained *in vitro,* we also tested the efficacy PMX/CPT-11 combination *in vivo* in CFPAC-1, MIA PaCa2 and PACA3 xenograft tumours established subcutaneously in athymic nude mice. Since the objective was to provide experimental basis for use in clinical trial as second-line salvage chemotherapy, we design to treat mice with PMX/CPT-11 after the GEM therapy and not as first-line therapy. Pemetrexed/CPT-11 combination showed the ability to further inhibit the cancer growth in the two lines partially responsive to GEM and to maintain the block of proliferation in the GEM full responsive line. Even if the *in vivo* results of PMX/CPT-11 treatment could appear quantitatively disappointing, starting from the presence of GEM pretreatment, they should be considered relevant.

The PMX/CPT-11 combination respects the four principles underlying the design of chemotherapy combination. First, each agent in a regimen has shown to be independently active against the pancreatic tumour. In fact, not only PMX, as above reported, has demonstrated to be active as single agent but CPT-11 also has demonstrated activity, although modest, in pancreatic cancer (Wagener *et al*, 1995; Klapdor and Fenner, 2000; Pizzolato and Saltz, 2003). Unfortunately, a recently reported phase III trial of GEM with or without CPT-11 revealed no survival benefit (Rocha Lima *et al*, 2004; Stathopoulos *et al*, 2006) but other combinations of CPT-11 should continue to be explored in an effort to further improve the treatment of this chemorefractory disease (Taieb *et al*, 2006). Secondly, each drug in this combination has an independent mechanism of action. In fact, PMX is an antimetabolite while CPT-11 is a selective DNA topoisomerase I inhibitor, targeting different steps along different biochemical pathways. Pavillard *et al* (1998) have shown an inverse relationship between thymidylate synthase activity and irinotecan-induced cleavable complex formation, which suggests a potential mechanism whereby synergy between PMX and CPT-11 might be expected to occur. Third, there is no crossresistance, at least *in vitro*, between PMX and CPT-11 and also among these two drugs and GEM. Fourth, PMX and CPT-11 have a different dose-limiting toxicity. Both the susceptibility to PMX/CPT-11 combination even in GEM-resistant lines and the evidence from previous works that *in vivo* full doses of PMX and CPT-11 are well tolerated in several small phase I and II trials in pretreated colorectal cancer patients (Grothey and Schmoll, 2001; Rowinsky *et al*, 2007) encourage the use of these two drugs in clinical experimental protocols as second-line treatment for pancreatic cancer.

In our study we also reported the evaluation of PMX combined with GEM, even if we looked for GEM-free protocol. The reason was that this combination represents a sort of reference point, since preclinical and clinical studies have shown synergy in a broad range of tumours including pancreatic cancer (Adjei *et al*, 2000; Giovannetti *et al*, 2004; Dy *et al*, 2005; Oettle *et al*, 2005). Consequently, the evidence of a major efficiency of the PMX–CPT-11 in comparison with the PMX–GEM should be considered as a further reason for its clinical testing. Of note, the phase III clinical study of PMX–GEM combination showed that the use of GEM intravenously over ∼30 min followed ∼90 min later by PMX does not improve survival in patients with unresectable locally advanced or metastatic pancreatic cancer (Kindler, 2002; Oettle *et al*, 2005). The absence of preclinical data of PMX–GEM combination specifically on pancreatic cancer was probably one of the cause of the failure of the phase III study. In fact, the use of the sequence was based on studies demonstrating synergistic cytotoxicity when GEM exposure precedes PMX exposure in HCT-8-cultured human colon cancer cell lines (Adjei *et al*, 2000) and on similar results obtained in LoVo, WiDr and LRWZ cells (Tesei *et al*, 2002). However, other reports proposed synergistic cytotoxicity for the opposite sequence PMX exposure followed 24 h later by GEM exposure (Tonkinson *et al*, 1999; Giovannetti *et al*, 2004). Our study suggested that the highest chemotherapeutic activity against MIA PaCa-2, PANC-1 and Capan-1 cells for PMX–GEM combination is observed with the sequence PMX → GEM, exactly the opposite of that used in clinical trial. Since the patterns of interaction with these two agents is cell line and tissue specific, the limited availability of preclinical data on PMX–GEM combination on pancreatic cancer likely hampered the rational design of the clinical study contributing to its failure.

To date, different options based on GEM are available for first-line treatment of pancreatic cancer (Burris *et al*, 1997; Reni *et al*, 2005). However, GEM-based chemotherapy yields a very limited disease control, and progression usually occurs within a few months after first-line treatment starts. In spite of progressive disease, about half of the patients maintain a good performance status and are willing to undergo further treatment. As no standard therapeutic option exists and scarce information on the impact on outcome of salvage therapy is available from the literature, studies attempting to widen the therapeutic armamentarium against this disease are warranted. Based on the result of our study, the PMX/CPT-11 association appears a promising GEM-free drug combination and we think that our results provide the experimental basis for its clinical testing as salvage chemotherapy in pancreatic cancer patients.

## Figures and Tables

**Table 1:** Effect of PMX : CPT-11 combination on cell recovery, cell cycle, caspase 3/7 activation: 48 h exposure

			**Cell recovery**			**Cell cycle (%)**					
**Cell line^a^**	**Source^b^**	**PMX–CPT-11**	**Cell number (× 10^6^)**	**%**	**% Caspase 3/7^+^**	**SubG1**	**G1**	**S**	**G2**	**Hyper G2**	**G2/G1**
MIAPaCa2	P	−	0.88		1.6	30	53	4	8	5	1.45
		+	0.68	77	2.9	51	27	11	7	4	0.27
											
PaCa3	P	−	1.7		6.9	17	60	3	14	7	0.23
		+	2.0	113	6.9	10	64	6	15	6	0.23
											
SK-PC 1	P	−	0.47		13	29	52	4	12	4	0.22
		+	0.23	50	18	47	27	11	11	5	0.41
											
PANC-2	P	−	1.95		8.9	4	51	15	23	9	0.45
		+	0.58	30	61.5	13	28	14	24	23	0.88
											
CFPAC1	M	−	0.56		19.8	19	34	3	17	28	0.49
		+	0.37	65	34.38	27	32	4	15	21	0.46
											
PC	M	−	1.17		24.8	6	39	5	28	22	0.72
		+	0.56	48	39.9	10	24	18	2	25	0.99
											
HS766T	M	−	0.23		37.4	24	39	7	17	15	0.43
		+	0.17	71	45.6	39	36	5	13	8	0.35
											
Capan-1	M	−	0.42		7.2	65	19	2	7	7	0.39
		+	0.25	60	5	71	11	5	6	6	0.53
											
T3M4	M	−	0.93		45	22	29	4	15	29	0.52
		+	0.49	53	61	50	27	6	7	10	0.26
											
A818-4	A	−	0.63		7.3	4	22	1	31	41	1.36
		+	0.70	111	11.8	8	22	8	27	37	1.20
											
Median		−	0.76		10.9	20.61	38.7	3.9	15.7	11.8	0.47
		+	0.52		26.1	33.11	27.2	6.7	13.7	8.9	0.43
											
*P* c			0.047		0.017	0.017	0.022	0.017	0.047	NS	NS

a Data reported are the mean of two experiments for each cell line.

b *P*=primary tumour; M=liver or lymph nodes metastasis; A=ascites.

c Wilcoxon Signed Rank test; NS=not statistically different.

PMX=pemetrexed; CPT-11=irinotecan.

**Table 2:** Effect of PMX : CPT-11 combination on cell recovery, cell cycle, caspase 3/7 activation and clonogenicity: 5 days after 48 h exposure

			**Cell recovery**			**Cell cycle (%)**						
**Cell line^a^**	**Source^b^**	**PMX–CPT-11**	**Cell number (× 10^6^)**	**%**	**%Caspase 3/7^+^**	**SubG1**	**G1**	**S**	**G2**	**Hyper G2**	**G2/G1**	**Clonogenicity (clone number)**
MIAPaCa2	*P*	−	0.56		1	27	51	6	11	6	0.22	5.25
		+	0.52	94	4.5	37	57	3	3	0	0.05	2
												
PaCa3	*P*	−	0.68		7.1	9	54	10	23	5	0.43	9.3
		+	0.76	111	16.9	20	31	11	27	11	0.88	7.9
												
SK-PC 1	*P*	−	0.22		0.8	15	57	9	12	8	0.21	2.5
		+	0.1	44	2.5	54	12	23	6	6	0.54	1.25
												
PANC-2	*P*	−	1.02		32	7	44	17	22	12	0.50	13
		+	0.08	8	57.7	55	8	12	10	14	1.18	2.5
												
CFPAC1	M	−	0.30		34.7	20	36	7	21	17	0.60	2.75
		+	0.19	56	31.8	47	10	11	19	13	1.96	1.31
												
PC	M	−	0.20		0.8	4	54	5	22	15	0.41	7.5
		+	0.10	50	7.4	15	13	25	24	25	1.85	3.5
												
HS766T	M	−	0.25		1.8	7	46	8	19	20	0.41	4.75
		+	0.15	60	7.6	16	19	23	18	25	0.95	1.25
												
Capan-1	M	−	0.12		3.7	59	21	5	8	7	0.36	4.25
		+	0.07	57	17.6	88	7	2	1	1	0.19	1.75
												
T3M4	M	−	0.45		15	16	40	2	17	24	0.43	3.75
		+	0.05	12	31	84	11	1	2	1	0.18	1
												
A818-4	A	−	0.24		8	6	47	13	23	10	0.49	4.25
		+	0.14	58	15	13	20	30	29	9	1.45	1.75
												
Median		−	0.28		5.4	11.6	46.7	7.7	20.2	10.8	0.41	4.5
		+	0.1		15.9	41.9	12.3	11.8	14.2	9.8	0.91	1.75
												
*P* c			0.009		0.005	0.005	0.007	NS	NS	NS	0.028	0.005

a Data reported are the mean of two experiments for each cell line.

b *P*=primary tumour; M=liver or lymph nodes metastasis; A=ascites.

c Wilcoxon Signed Rank test; NS=not statistically different.

PMX=pemetrexed; CPT-11=irinotecan.
